# Targeting Aggressive Pituitary Adenomas at the Molecular Level—A Review

**DOI:** 10.3390/jcm11010124

**Published:** 2021-12-27

**Authors:** Benjamin Voellger, Zhuo Zhang, Julia Benzel, Junwen Wang, Ting Lei, Christopher Nimsky, Jörg-Walter Bartsch

**Affiliations:** 1 Department of Neurosurgery, University Hospital Marburg, Baldingerstr., 35033 Marburg, Germany; zhangzhuo1991@hust.edu.cn (Z.Z.); j.benzel@kitz-heidelberg.de (J.B.); wangjunwen1003@gmail.com (J.W.); christopher.nimsky@med.uni-marburg.de (C.N.); jbartsch@med.uni-marburg.de (J.-W.B.); 2 Department of Neurosurgery, Tongji Hospital, Tongji Medical College of Huazhong University of Science and Technology, Wuhan 430030, China; tlei@tjh.tjmu.edu.cn; 3 Deutsches Krebsforschungszentrum (DKFZ) Heidelberg, Im Neuenheimer Feld 280, 69120 Heidelberg, Germany

**Keywords:** pituitary adenomas, adjuvant treatment, hormone secretion, invasiveness, molecular biology, proliferation

## Abstract

Pituitary adenomas (PAs) are mostly benign endocrine tumors that can be treated by resection or medication. However, up to 10% of PAs show an aggressive behavior with invasion of adjacent tissue, rapid proliferation, or recurrence. Here, we provide an overview of target structures in aggressive PAs and summarize current clinical trials including, but not limited to, PAs. Mainly, drug targets in PAs are based on general features of tumor cells such as immune checkpoints, so that programmed cell death 1 (ligand 1) (PD-1/PD-L1) targeting may bear potential to cure aggressive PAs. In addition, epidermal growth factor receptor (EGFR), mammalian target of rapamycin (mTOR), vascular endothelial growth factor (VEGF), fibroblast growth factor (FGF) and their downstream pathways are triggered in PAs, thereby modulating tumor cell proliferation, migration and/or tumor angiogenesis. Temozolomide (TMZ) can be an effective treatment of aggressive PAs. Combination of TMZ with 5-Fluorouracil (5-FU) or with radiotherapy could strengthen the therapeutic effects as compared to TMZ alone. Dopamine agonists (DAs) are the first line treatment for prolactinomas. Dopamine receptors are also expressed in other subtypes of PAs which renders DAs potentially suitable to treat other subtypes of PAs. Furthermore, targeting the invasive behavior of PAs could improve therapy. In this regard, human matrix metalloproteinase (MMP) family members and estrogens receptors (ERs) are highly expressed in aggressive PAs, and numerous studies demonstrated the role of these proteins to modulate invasiveness of PAs. This leaves a number of treatment options for aggressive PAs as reviewed here.

## 1. Introduction

Pituitary adenomas (PAs) originate from the anterior lobe of the pituitary gland and account for about 15 per cent of all intracranial neoplasms [1]. The overwhelming majority of PAs are benign, albeit up to 35 per cent of them exhibit locally invasive behavior [2]. Invasion of PAs into the cavernous sinuses can be classified according to Hardy or Knosp [3,4].

The recently coined term “aggressive” PA refers to a clinically defined subset of tumors that are highly proliferative or invasive and resistant to all standard treatments [5,6,7]. Depending on the level of specialization of the treatment center and on the exact definition applied, such aggressive tumors may account for up to 10 per cent of cases [6]. Very rarely, in about 0.2 per cent of cases, pituitary carcinomas, i.e., metastasizing tumors of the anterior lobe of the pituitary gland, are encountered [8].

PAs may lead to increased hormone secretion, hormonal insufficiency of the pituitary gland, facial pain, and impaired function of the visual apparatus, i.e., double vision, visual field cuts, loss of visual acuity, and even blindness [1]. Endocrine malfunction or neurological deficiencies are indications to treat PAs [1].

Surgical resection of the tumor mass is the first line treatment for all PAs except prolactinomas [1]. Patients with prolactinomas that do not respond to medical treatment or who experience strong side effects after medical treatment should also be treated surgically [1]. Up to 90 per cent of PAs can be resected safely using a transsphenoidal approach, guided by fluoroscopy and with the help of microsurgical techniques [1]. Pronounced suprasellar asymmetry and retrosellar or subfrontal growth of the tumor may render a transcranial approach more feasible [1]. The surgical treatment of PAs is continuously being refined due to the advancement of intraoperative visualization techniques, such as endoscopy, intraoperative computerized tomography, intraoperative magnetic resonance imaging, and neuronavigation [9,10,11].

Radiation therapy is usually applied with remnants or recurrence of PAs at inoperable sites [1]. The inherent risks of radiation therapy are rarely encountered. They comprise tumor induction and damage to surrounding healthy tissues [1].

Obtaining clinical control of aggressive PAs remains, however, a largely unsolved problem. While recently collected evidence demonstrates that 47 percent of aggressive PAs respond well to the administration of the alkylating agent Temozolomide (TMZ) [6], the majority of these life-threatening tumors still escape best medical practice. This review aims to comprise and discuss other potential options to target aggressive PAs at the molecular level.

## 2. Emerging Targeted Treatment Strategies at the Molecular Level

**Figure 1:**
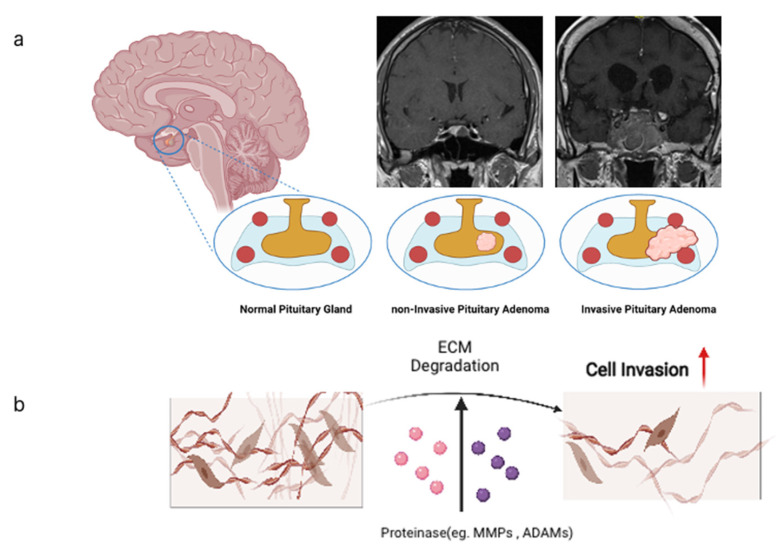
(**a**) Pituitary gland located in the sella turcica shown in coronal sections as normal pituitary gland, benign pituitary adenoma, and as invasive pituitary adenoma. Internal carotid arteries adjacent to the pituitary are depicted as red circles. Cavernous sinuses are depicted in light blue. Note that aggressive PAs tend to circumvent the arteries. (**b**) Cellular mode of invasion into the brain tissue depends on degradation of extracellular matrix (ECM) molecules by proteinases of the metzincin family, namely MMPs and ADAMs. All images produced with Biorender (https://biorender.com (accessed on 6 November 2021)).

**Figure 2:**
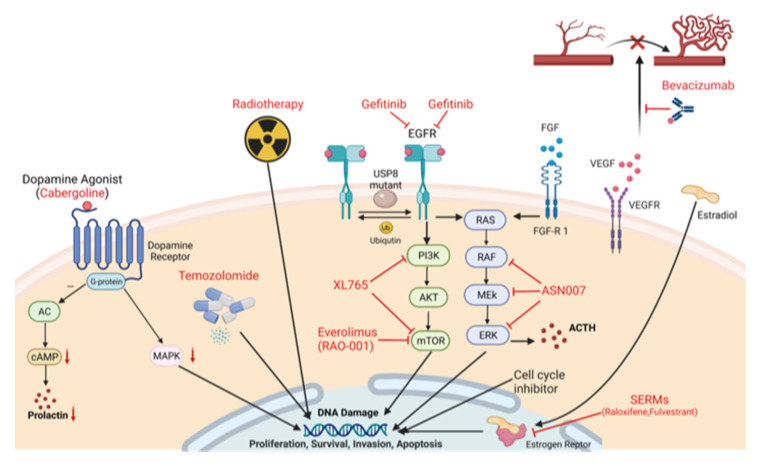
Schematic overview of target proteins and pathways related to the treatment of pituitary adenomas. As general therapeutic approaches, VEGF inhibition with Bevacizumab, EGFR inhibition with gefitinib, the Raf/MEK/ERK pathway by inhibitors such as ASN007, mTOR pathway inhibition with Everolimus and XL-765 as well as chemotherapy with radiation or temozolomide are discussed. As a more specialized therapy, application of dopamine agonists such as Cabergoline and anti-estrogens is the subject of intense research.

Treatment strategies can be targeted to treat either pituitary adenomas in their localized form or as their aggressive counterparts when tumor mass is invading the surrounding tissue (Figure 1). An overview on the target proteins and their related pathways relevant for PAs is shown (Figure 2), and the relevant pathways are introduced in the following chapters.

### 2.1. Dopamine Agonist (DA) Treatment, and Treatment of Excess Hormone Secretion

Dopamine modulates hormone secretion in PAs. Based on this, medication with dopamine agonists (e.g., Cabergoline, Bromocriptine) is the first line chemotherapy treatment option for patients with prolactinomas [1] due to their high expression levels of dopamin 2 receptors (D2R). However, other PA subtypes also express D2R in varying degrees, so that dopamine agonists may also serve as treatment options for these tumor types including growth hormone (GH)-secreting PAs [1]. Somatostatin analogues (e.g., Octreotide) and the GH receptor antagonist Pegvisomant may help in cases of GH-secreting PAs that are refractory to other treatment options [1,12]. In adrenocorticotropic hormone (ACTH)-secreting PAs, adrenostatic drugs (e.g., Ketoconazole, Metyrapone, Mifepristone, Osilodrostat) may lower excessive, symptomatic glucocorticoid levels [1,13,14]. Since ACTH-secreting PAs also express somatostatin receptors (SSTRs), treatment of these tumors with somatostatin analogues like Pasireotide is a valid option [13,15,16]. Dopamine receptor (DR) expression in ACTH-secreting PAs has been described [17]. While several case reports have demonstrated that ACTH-secreting PAs may respond to treatment with Cabergoline [18,19,20,21,22,23], the results in larger cohorts of patients bearing such tumors still remain controversial [24,25]. However, administration of dopamine agonists is worth considering as an option to treat these tumors [13,14], and a clinical trial on Cabergoline treatment in corticotroph PAs (Mumbai, India (https://clinicaltrials.gov (accessed on 6 November 2021)) [26]; trial identifier: NCT00889525; Table 1) has been initiated.

The existence of D2R in non-functioning PAs (NFPAs) [27,28,29] justifies to consider DA treatment as an option in residual or refractory cases of NFPAs [1]. This option has been evaluated in several clinical studies [30,31,32]. Based on these results, a Brazil team launched a randomized clinical trial to examine the efficacy of the DA Cabergoline in the treatment of NFPA residuals after transsphenoidal surgery (São Paulo, Brazil [26]; trial identifier: NCT03271918). Batista et al. drew the conclusion that Cabergoline was effective in residual NFPAs [33]. Cabergoline as a treatment for refractory NFPAs is currently being investigated in a Scandinavian multicenter study (Oslo and Trondheim, Norway; Gothenburg, Sweden [26]; trial identifier: NCT02288962; Table 1).

### 2.2. Epidermal Growth Factor-Receptor (EGFR) Inhibition

Epidermal growth factor (EGF), acting through the EGF-receptor (EGFR), is a potent modulator of cell proliferation and differentiation in a wide variety of cell types. Expression of EGF and EGFR has been detected in both the normal pituitary gland and in PAs [34], as well as in rat and mouse PA cell lines [35,36,37]. PRL secretion and gene expression, tumor size, and tumor invasion are related to EGFR in human prolactinomas and in animal models [38,39]. In EGF gene transfected mice, PRL secretion and tumor size have been downregulated [40]. To attenuate EGFR pathway effects in cancers, EGFR Tyrosine kinase inhibitors (TKIs) that bind the tyrosine kinase domain of EGFR specifically and inhibit its activity are widely used [41,42].

Lapatinib is an ErbB1-epidermal growth factor receptor (EGFR)/ErbB2 or human EGFR2 tyrosine kinase inhibitor that has proven efficacy in breast cancer and other solid tumors [43]. In a recent study (Boston, USA [26]; trial identifier: NCT00939523; Table 1) on prolactinomas that were resistant to dopamine agonist therapy, lapatinib was explored in four patients with oral lapatinib at a dose of 1250 mg daily for up to 6 months. Although no patient reached the endpoint criteria (40% reduction of tumor volume assessed by MRI), three patients showed a stable disease with a 16.8% reduction of tumor diameter in one case and with a 6% increase in 2 cases, while the remaining patient had a progressive disease. In conclusion, lapatinib was well tolerated and caused transaminitis in two patients, grade 2 rash in two patients and grade 1 asymptomatic bradycardia in two patients. The investigators drew the conclusion that Lapatinib may be effective in patients with aggressive prolactinomas [44].

At the same time, the ubiquitin specific peptidase 8 (USP8), another promising downstream effector of EGFR, is a potential molecular target in pituitary adenomas refractory to standard treatment. Gefitinib, an EGFR inhibitor, showed efficacy in ACTH-secreting PA cells of human origin, with a mutation of the USP8 gene present in the majority of cases [45,46]. A clinical trial investigating the treatment of patients with USP8-mutated ACTH-secreting PAs has been initiated (Shanghai, China [26]; trial identifier: NCT02484755; Table 1).

### 2.3. Estrogen Receptor Modulation

In humans, two intracellular subtypes of estrogen receptors (ERs) are known: ER1, and ER2. While the DNA binding domains of both receptors share a similar structure, their hormone binding domains differ. Tissues with ER1 expression include bone, brain, epididymis, hypothalamus, mammary gland, ovary, pituitary, prostatic gland, testes, and uterus. Tissues with ER2 expression include bone marrow, brain, intestine, ovary, prostatic gland, salivary glands, and testes [47]. The binding of ERs to hormone responsive elements (HREs) in the promotor region of target genes induces the genomic effects of ERs, with HREs showing partial differences between ER subtypes. Modulation of other transcriptional factors through ERs and primarily non-genomic effects of ERs have also been observed [48]. ER ligands may either modulate the activity of the receptor (selective estrogen receptor modulators (SERMs)) or bind to the receptor and then degrade it (selective estrogen receptor degraders (SERDs)) [49]. Several SERMs approved to treat other conditions in humans have drawn the attention of scientists exploring potential salvage therapies for aggressive PAs.

The experimental use of the SERM Bazedoxifene in rats for up to 2 years led to a significant increase in survival and to significantly less tumors of the mammary and pituitary glands [50]. While in vitro experiments indicate that Bazedoxifene significantly decreases survival, invasiveness, and expression of invasion-related proteases in rodent PA cells [51], clinical experience in treating aggressive PAs with Bazedoxifene has not yet been reported.

Walker et al. [52] reported in 1996 on a case of hemorrhagic transformation of a GH-secreting macroadenoma of the pituitary gland in a woman with primary infertility who had received the SERM Clomifene. At that time, the authors assumed that Clomifene may have induced pituitary apoplexy via an indirect increase in portal blood gonadotropic releasing hormone levels. Taking into account results from recent in vitro investigations on the impact of Clomifene on rodent PA cell survival [51], a direct effect of the drug on PA cells with subsequent pituitary apoplexy is also conceivable.

In 2004, Dimaraki et al. [53] reported that a daily dose of 2 × 60 mg of the SERM raloxifene reduced serum insulin-like growth factor (IGF)-1 levels by a small but statistically significant amount in acromegalic men. In their hands, the rather short course of treatment with Raloxifene (up to 6 weeks) did not result in relevant changes of the clinical presentation or in serum levels of GH, prolactin, and testosterone. The authors supposed a direct impact of the drug on hepatic metabolism rather than on the hypothalamic-pituitary axis. In a recently published retrospective study, Choudhary et al. [54] investigated the efficacy of a daily dose of 60 mg raloxifene plus dopamine agonists for up to 6 months in patients with prolactinomas whose hormone levels did not normalize despite dopamine agonist treatment. In 10 out of 14 patients, serum prolactin levels were reduced by 25.9 per cent on average. In 2 out of 10 patients, normoprolactinemia was achieved. Raloxifene has been found to decrease survival, invasiveness, and expression of invasion-related proteases in rodent PA cells in vitro [51].

The combination of the SERM tamoxifene with dopamine agonists to treat PAs that are refractory to standard therapies had been tried decades ago, with results that many clinicians considered discouraging [55,56]. Tamoxifene plus dopamine agonists led, however, to long-term tumor control in a recently reported case of an aggressive PA which, in the absence of ER1 expression, exhibited markedly high ER2 expression [57].

The results from in vitro experiments in rodent PA cell lines and the evidence collected in humans indicate that SERMs may serve as a salvage therapy in aggressive PAs [51,58,59,60]. While membrane-bound G protein-coupled ERs are apparently involved in rodent PA cellular signal transduction [61,62,63,64], the potential clinical implications of these findings remain to be elucidated.

### 2.4. Mammalian Target of Rapamycin (mTOR) Inhibition

The phosphoinositide 3-kinase(PI3K)/protein kinase B(AKT)/mTOR signaling pathway is involved in tumor cell metabolism, apoptosis, and proliferation [65]; it has been demonstrated to be overactivated in pituitary tumors [66]. AKT expression and phosphorylation have also been reported to be elevated in PAs as compared to normal pituitary gland tissue [67]. In one study, mTOR pathway activation was noted in 43% of all PA patients’ samples, and in 71% of samples of growth hormone (GH) secreting tumors [68]. mTOR forms two major protein complexes with other protein partners, namely mTOR complex-1 (mTORC1) and mTOR complex-2 (mTORC2). mTORC1 is formed by mTOR and Regulatory-associated protein of mTOR (RAPTOR), whose expression was confirmed in PAs and is correlated with invasion and tumor growth [69]. Everolimus is a first-generation inhibitor of mTOR that binds to mTOR allosterically in a complex with FK506-binding protein 12 (FKBP12) thereby inhibiting mTORC1 activity [70]. Everolimus has been approved for the treatment of advanced renal cell carcinoma [71]. Gorshtein, Zatelli and colleagues [72,73,74] confirmed that Everolimus helps to reduce cell viability both in cell lines and primary human PA cells, where the PI3K/AKT/mTOR signaling pathway is involved in the regulation of GH secretion. Donovan et al. [75] reported on widely metastatic ACTH-secreting pituitary carcinoma in a patient carrying a serine/threonine kinase 11 (STK11) (F298L) mutation in the mTOR pathway, and Everolimus monotherapy stabilized the disease for more than 6 months. Zhang et al. [76] combined Everolimus with Cabergoline that led to decreased PRL levels and tumor regression after 5 months. In the future, with the wide use of next generation-sequencing (NGS), refractory pituitary tumor patients could be diagnosed for the PI3K/AKT/mTOR signaling pathway and, if positive, Everolimus could be one potent treatment option.

### 2.5. Metalloprotease Inhibition

The human matrix metalloproteinases (MMPs) family comprises about 30 members, including soluble secretory proteins and cell-membrane-associated proteins [77]. All members have a conserved zinc-binding motif within the catalytic domain; it is produced initially in an inactive form, and is converted into an active form through proteolytic removal of the pro-domain [78]. Active forms of MMPs are well known to degrade various components of extracellular matrices, including collagens, fibronectin, and laminins, and to activate other MMPs [78].

A disintegrin and metalloproteinases (ADAMs) are membrane-spanning cell–cell and cell–matrix interactive proteins. They are uniquely characterized as having a disintegrin domain and a metalloproteinase-like domain within their molecules [79]. Some ADAMs have a degradative activity on extracellular matrix (ECM) components such as fibronectin, and some are known to cleave membrane proteins so that they are removed from the cell surface.

Thus, during carcinogenesis, MMPs and ADAMs participate in several interactions with the tumor microenvironment involving extracellular matrix (ECM), growth factors and cytokines associated with the ECM and surrounding cells. MMPs and ADAMs play important roles in cell proliferation, apoptosis, angiogenesis, invasion, migration, and epithelial to mesenchymal transition (EMT) [80]. For these reasons, MMPs have been considered as potential diagnostic and prognostic biomarkers in many types and stages of cancer [81]. However, of all MMP and ADAM family members, only some bear the potential to interfere with cancer development. In 2005, Liu et al. [82] confirmed by immunohistochemistry that, in human samples, MMP-2 expression is higher in PAs invading the cavernous sinuses as compared to non-invasive PAs. Malik et al. [83] found evidence that MMP-2 may manipulate oncogenic functions of the pituitary tumor transforming gene (PTTG). By contrast, Beaulieu et al. [84] have tested 12 normal pituitary gland samples and 28 human pituitary tumor tissue samples by Western blot and drew the conclusion that MMP-1, -2, and -3 expression levels had no correlation with tumor invasiveness. MMP-9 is another protein that impacts tumor invasion and recurrence in PA cell lines resp. human PA samples [85,86,87,88]. By contrast, Knappe et al. [89] found no correlation between MMP-9 expression and tumor invasion. Apart from MMP-2 and MMP-9; ADAM12, MMP-14, and ADAM10 were also found to promote migration and to be associated with invasion in PAs [90,91].

MMP and ADAM family members have been thoroughly investigated in the past decades. There are numerous metalloprotease inhibitors (MPIs) directed either specifically against a limited number of MMPs or acting within a broader range. Preclinical studies testing the efficacy of MMP suppression in tumor models were so compelling that synthetic MPIs were rapidly developed and routed into human clinical trials. However, the results of these trials have so far been disappointing [92]. Some drugs that are approved for other clinical applications, like the SERM Clomiphene [51,60], may inhibit PA cell invasiveness by affecting MMP signaling pathways.

### 2.6. Peptide Receptor Radionuclide Therapy (PRRT)

Peptide receptor radionuclide therapy denotes a systemic radiotherapy that allows targeted delivery of radionuclide to tumors that show high expression of somatostatin receptors, usually by 2 well established radiopeptides serving as ligands of somatostatin receptors (SSTRs) in neuroendocrine tumors. These two radiopeptides are ^90^Y-DOTATOC and ^177^Lu-DOTATATE, so that cytotoxic doses of radionuclides may be linked to SSTR ligands in order to treat tumors with SSTR expression. A phase III clinical trial has demonstrated the efficacy of PRRT in advanced stages of intestinal neuroendocrine tumors [93]. PRRT with cytotoxic labeled ligands may be helpful even in the treatment of relatively small tumors, as long as they exhibit marked SSTR expression [94]. The fact that certain PAs overexpress SSTR [95] renders PRRT a promising option in these tumors. A case reported by Komor et al. in 2014 demonstrated that the two radiopeptides were successful in a patient with non-functional pituitary adenoma (WHO grade II) by stabilizing the disease [96]. Giuffrida et al. reported in 2019 on their single center-experience with PRRT for aggressive PAs. In their hands, 1 out of 3 patients was treated successfully with PRRT after failure of conventional treatment [97].

### 2.7. Vascular Endothelial Growth Factor (VEGF) Inhibition

Notwithstanding all approaches to classify PAs, these tumors are basically solid, and their expansion thus depends on neovascularization through angiogenesis [98,99]. This renders vascular endothelial growth factor (VEGF) and its receptors VEGFR-1, VEGFR-2 potentially important in the treatment of aggressive PAs. As compared to normal pituitary gland tissue, PAs can express higher levels of VEGF [100] thus qualifying an anti-VEGF treatment using bevacizumab, a monoclonal antibody against VEGF. However, there are only a few cases for which anti-VEGF therapy has been reported as an alternative to current non-targeted therapies. For instance, bevacizumab has been applied in a few cases of aggressive PAs resp. pituitary carcinomas, with a wide range of outcomes. One such example described a patient with 7 surgeries, radiation therapy and three courses of TMZ whose disease was stabilized upon bevacizumab administration for 26 months [101]. VEGFR-2 is the principal mediator of the VEGF-induced signal pathway. Thus, inhibition of VEGFR-2 could be another promising strategy to down-regulate tumor angiogenesis [102]. Apatinib (YN968D1) is a small-molecule antiangiogenic agent that selectively inhibits VEGFR-2. Wang et al. [103] reported on a successful case with a combination of Apatinib and Temozolomide in a case of recurrent invasive PA, resulting in 31.5 months of recurrence-free survival.

### 2.8. Fibroblast Growth Factor (FGF)

Tumor associated fibroblasts (TAFs) have been shown to contribute to the aggressiveness of PAs [104]. Secretion of cytokines, among them FGF-2, was shown to increase invasiveness of pituitary adenoma cells and induce EMT. This secretion is a consequence of somatostatin receptor stimulation in TAFs, so that Pasireotide, a SSTR antagonist, reduces cytokine release from TAFs and thereby invasiveness of PA cells. FGF-2 stimulation can be mediated via the rapidly accelerated fibrosarcoma (Raf)/mitogen-activated protein kinase kinase (MEK)/extracellular signal-regulated kinase (ERK) pathway similar to EGFR stimulation. Up to now, no clinical data are available targeting this particular pathway in PAs. 

### 2.9. Raf/MEK/ERK Pathway

In addition to the induction of this pathway by EGF ligands and by FGF receptor stimulation, it has been shown that the Leucine-rich repeats and immunoglobulin-like domains protein 1 (LRIG1) can suppress the biological function of PAs by attenuation of the PI3K and rat sarcoma (Ras)/Raf/MEK/ERK pathway. Over-expression of LRIG1 in nude mice resulted in reduced PA proliferation and invasion and in enhanced PA apoptosis [105].

## 3. Non-Targeted Treatment

### 3.1. TMZ and 5-Fluorouracil (5-FU) Treatment

TMZ is an alkylating agent with a good bioavailability of almost 100% following oral administration. The European Society of Endocrinology (ESE) has recommended TMZ for the treatment of aggressive PAs and pituitary carcinomas after failure of surgery, conventional medical treatments, and radiotherapy [6]. However, a positive effect of TMZ has been observed in only 47 per cent of cases [6]. Thus, the combination of TMZ with other effective drugs is worth considering in aggressive PAs [106]. In 2011, Thearle et al. [107] reported on a patient with an aggressive corticotroph PA resp. pituitary carcinoma who benefitted from the combination of Capecitabine (a prodrug of 5-FU) and TMZ. A case series reported by Zacharia et al. [108], including 4 patients with ACTH-secreting PAs refractory to other therapies, indicated that the combination of TMZ with 5-FU might yield a higher success rate as compared to treatment with TMZ alone. A respective clinical trial was initiated (New York, NY, USA [26]; trial identifier: NCT03930771; Table 1).

### 3.2. TMZ and Radiotherapy

TMZ bears additional potential as a radiosensitizer [109]. In patients with newly diagnosed glioblastoma multiforme, postoperative treatment with concomitant TMZ and radiotherapy has proven safe and efficient at the highest level of evidence, thus being the standard therapy for these tumors since more than a decade using the Stupp protocol 75 mg/m^2^ daily for 6 weeks with parallel radiotherapy, followed by dosing 150–200 mg/m^2^ for 5 days every 28 days [110]. The ESE [106] considers concomitant TMZ and radiotherapy as promising also in cases of aggressive PAs, which will be further investigated in a clinical trial in the near future (Beijing, China [26]; trial identifier: NCT04244708; Table 1).

## 4. Checkpoint Inhibition

In rapidly growing tumors, genomic alterations may lead to dysfunction of deoxyribonucleic acid (DNA) repair proteins (mismatch repair deficiency (MMRD)). MMRD frequently results in hypermutation, i.e., stepwise accumulation of insertions, deletions and alterations of short DNA sequences (microsatellites). A comparison of microsatellite length between tumor and healthy tissue allows to detect microsatellite instability (MSI). MSI is considered a marker for hypermutation resp. MMRD [111].

On the one hand, tumor cells with MMRD have a high tumor mutational burden (TMB), thus presenting on their surface a variety of neo-antigens to the immune system. Lymphocytic cell populations that have invaded the tumor tissue are called tumor infiltrating lymphocytes (TILs). In breast cancer, TILs are comprised primarily of cytotoxic (CD8+) and helper (CD4+) T-lymphocytes, and a smaller proportion of B- and NK cells [112]. Along with other mononuclear cells, they form the tumor immune microenvironment and play a critical role in tumor progression. It has been shown that TILs exist in PAs [113,114,115,116]. Programmed cell death-1 (PD-1) is an immune checkpoint that is expressed predominantly by T-lymphocytes [117].

On the other hand, immunosuppressive checkpoint ligands and cytokines, as synthesized by tumor cells, attenuate the immune response by binding of the checkpoint ligand PD-L1, which has been described in human PAs [116,118,119,120,121].

**Figure 3:**
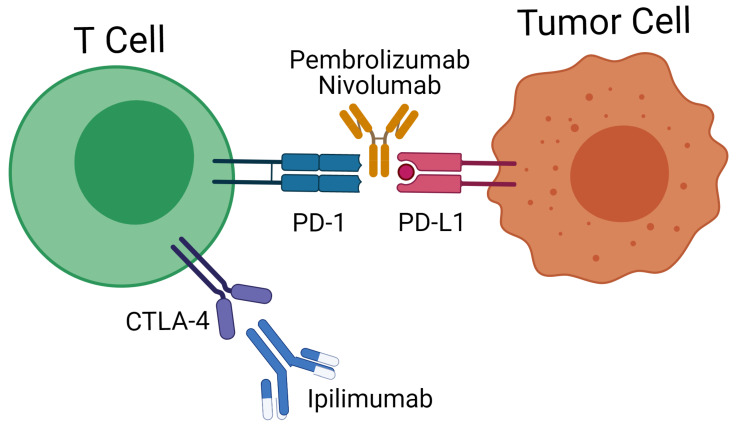
Immune checkpoint inhibition using anti-PD-L1 antibodies in clinical use such as nivolumab and pembrolizumab. In addition, anti-CTLA4 therapy involving ipilimumab has also been considered in PA treatment.

Checkpoint inhibitors (e.g., Ipilimumab, Nivolumab, Pembrolizumab) are monoclonal antibodies that prevent checkpoint ligands to interact with the respective surface proteins (checkpoints) of the T-lymphocytes, so that the neo-antigens may elicit an adequate response of the immune system against the tumor cells (Figure 3).

Inhibiting the PD-1 pathway has been proven to be highly effective in lung cancer [122] and melanomas [123] as compared to traditional chemotherapy. CTLA4 is a T-cell located protein that can turn down the immune response in the early phase of tumor development. Thus, using a CTLA4 antagonist could elicit anti-tumoral effects in the early phase of T cell activation (Figure 3) [123].

The idea to combine Ipilimumab and Nivolumab in treating aggressive PAs is substantiated [121,124] and has already prompted clinical investigation (New Jersey and New York, NY, USA [26]; trial identifier: NCT04042753 and Bethesda, USA [26]; trial identifier: NCT02834013; Table 1). This particular combination of checkpoint inhibitors may, however, yield considerable side effects [125]. Furthermore, not all patients eligible for checkpoint inhibition actually benefit from such a treatment. In a phase II clinical trial for Pembrolizumab (Houston, USA [26]; trial identifier: NCT02721732; Table 1), Majd et al. [126] included 4 patients with refractory PAs, 2 of whom had partial radiographic and hormonal responses after initiation of Pembrolizumab.

There is evidence that the tumor microenvironment, rather than the origin of the tumor, determines the efficacy of immune checkpoint inhibition [127,128,129,130]. Hypermutation in aggressive tumors of the anterior pituitary gland has been observed [131]. Furthermore, tumor treatment with alkylating agents, such as TMZ, may not only induce cell death, but also lead to hypermutation in vital tumor cells [132,133].

With regard to the aforementioned drug targets and immune processes occurring in invasive pituitary adenomas, a number of clinical trials are in progress to address the efficacy of these drugs for the treatment of aggressive PAs. These trials are listed in Table 1.

## 5. Conclusions

We reviewed the most recent discoveries on treatment of aggressive pituitary adenomas at the molecular level. By reviewing different molecular cues for the observed aggressiveness of PAs, we provide a rationale for clinical interventions in aggressive pituitary adenomas.

## Figures and Tables

**Table 1:** List of clinical trials related to the treatment of aggressive PAs (https://clinicaltrials.gov [26] (accessed on 6 November 2021)).

Mechanism of Action	Intervention(s)	Trial Identifier	Study Title	Recruitment Status	Condition	Phase	Number of Enrolled Patients	Study Type	Primary Purpose
checkpoint inhibition	Ipilimumab, Nivolumab	NCT04042753	Nivolumab and Ipilimumab in People With Aggressive Pituitary Tumors	recruiting	pituitary tumor	II	21 *	clinical trial	treatment
checkpoint inhibition	Ipilimumab, Nivolumab	NCT02834013	Nivolumab and Ipilimumab in Treating Patients With Rare Tumors	recruiting	pituitary carcinoma	II	818 *	clinical trial	treatment
checkpoint inhibition	Pembrolizumab	NCT02721732	Pembrolizumab in Treating Patients With Rare Tumors That Cannot Be Removed by Surgery or Are Metastatic	active, not recruiting	pituitary tumor	II	202	clinical trial	treatment
dopamine agonist treatment	Cabergoline	NCT03271918	Cabergoline in Nonfunctioning Pituitary Adenomas (NFPA)	completed	NFPA	III	140	clinical trial	treatment
dopamine agonist treatment	Cabergoline	NCT02288962	Dopamine Agonist Treatment of Non-functioning Pituitary Adenomas	recruiting	NFPA	III	60 *	clinical trial	treatment
dopamine agonist treatment	Cabergoline	NCT00889525	Study of Cabergoline in Treatment of Corticotroph Pituitary Adenoma	completed	Cushing’s disease	III	unknown	clinical trial	treatment
epidermal growth factor receptor (EGFR) inhibition	Lapatinib	NCT00939523	Targeted Therapy With Lapatinib in Patients With Recurrent Pituitary Tumors Resistant to Standard Therapy	completed	pituitary tumor	II	9	clinical trial	treatment
epidermal growth factor receptor (EGFR) inhibition	Gefitinib	NCT02484755	Targeted Therapy With Gefitinib in Patients With USP8 **-mutated Cushing’s Disease	unknown	Cushing’s disease	II	6 *	clinical trial	treatment
interference with deoxyribonucleid acid (DNA) replication	Temozolomide, Radiotherapy	NCT04244708	The Effect of Chemoradiotherapy in Patients With Refractory Pituitary Adenomas	not yet recruiting	pituitary tumor	II	150 *	clinical trial	treatment
interference with deoxyribonucleid acid (DNA) replication	Temozolomide, Fluorouracil	NCT03930771	Capecitabine and Temozolomide for Treatment of Recurrent Pituitary Adenomas	terminated	pituitary tumor	II	1	clinical trial	treatment

* estimated enrollment; ** ubiquitin specific peptidase 8.

## Data Availability

Not applicable.
